# Influence of European Beech (Fagales: Fagaceae) Rot Hole Habitat Characteristics on Invertebrate Community Structure and Diversity

**DOI:** 10.1093/jisesa/ieab071

**Published:** 2021-09-23

**Authors:** Jordan P Cuff, Fredric M Windsor, Emma C Gilmartin, Lynne Boddy, T Hefin Jones

**Affiliations:** 1 School of Biosciences, Cardiff University, Museum Avenue, Cardiff CF10 3AX, UK; 2 School of Natural and Environmental Science, Newcastle University, Agriculture Building, Newcastle upon Tyne NE1 7RU, UK; 3 Woodland Trust, Kempton Way, Grantham, Lincolnshire NG31 6LL, UK

**Keywords:** forest entomology, conservation, community ecology, habitat management

## Abstract

Hollows of veteran trees (i.e., rot holes) provide habitat for many rare and threatened saproxylic invertebrates. Rot holes are highly heterogeneous, particularly in terms of substrate and microclimate conditions. There is, however, a dearth of information regarding the differences in biological communities inhabiting rot holes with different environmental conditions. Invertebrates were sampled from European beech (*Fagus sylvatica*) rot holes in Windsor, Savernake, and Epping Forests (United Kingdom). For each rot hole, physical and environmental conditions were measured, including tree diameter, rot hole dimensions, rot hole height, substrate density, water content, and water potential. These parameters were used to assess the influence of environmental conditions and habitat characteristics on invertebrate communities. Rot hole invertebrate communities were extremely diverse, containing both woodland generalist and saproxylic specialist taxa. Large variation in community structure was observed between rot holes and across woodlands; all sites supported threatened and endangered taxa. Environmental conditions in rot holes were highly variable within and between woodland sites, and communities were predominantly structured by these environmental conditions. In particular, turnover between invertebrate communities was linked to high β-diversity. The linked heterogeneity of environmental conditions and invertebrate communities in rot holes suggests that management of deadwood habitats in woodlands should strive to generate environmental heterogeneity to promote invertebrate diversity. Additional research is required to define how management and conservation activities can further promote enhanced biodiversity across rot holes.

Rot holes, or tree hollows and cavities, can provide stable and long-lasting habitats for many organisms (Müller et al. 2014), including saproxylic invertebrates (Ódor et al. 2006, Abrego et al. 2015, Horák 2017) which depend on deadwood during at least one stage of their life cycle. Rot hole invertebrate fauna spans a broad taxonomic and functional diversity (Quinto et al. 2014, Taylor and Ranius 2014, Zheng et al. 2016) including many of Europe’s most threatened species (Gouix et al. 2009, Gough et al. 2014, Cálix et al. 2018). Taxa that occupy rot holes are either habitat specialists, or form communities that are distinct from those found in other woodland habitats (Muller et al. 2002, Taylor and Ranius 2014).

Previous intensive woodland management has led to a significant loss of large old trees and their associated microhabitats in many woodland systems (Lindenmayer et al. 2014). As a result, current sustainable woodland management aims to reinstate these deadwood habitats (Lindenmayer and Laurance 2017). Management of rot hole habitats, including the maintenance of inter-hole heterogeneity, is fundamental for the conservation and restoration of woodland biodiversity (Rotherham 2013, Siitonen and Ranius 2015, Horák 2017, Hall 2018) and saproxylic taxa not found in other woodland habitats (Müller et al. 2014, Quinto et al. 2014, Schauer et al. 2018). The relationship between rot hole conditions and associated biological communities is, however, poorly understood (Taylor and Ranius 2014), making it difficult to action appropriate measures.

Using family-level analyses of whole invertebrate communities, this study aims to investigate rot hole invertebrate community structure and its relationship with a range of microhabitat characteristics. It was hypothesized that rot holes contain diverse invertebrate communities that are: (i) significantly different from one another within and between woodland sites; (ii) related to physical habitat characteristics such as tree diameter and rot hole height; and (iii) different from one another across gradients of microhabitat conditions (e.g., rot density and water content).

## Methods

### Sample Collection and Sorting

Samples were collected between February and July 2016 from three ancient woodlands in southern England, United Kingdom: Epping Forest (51°39′24.7′′N, 0°02′33.0′′E), Savernake Forest (51°23′39.4′′N, 1°41′07.1′′W), and Windsor Forest (51°26′02.5′′N, 0′38′37.2′′W). All woodlands are Sites of Special Scientific Interest (SSSI) notified under Section 28 of the Wildlife and Countryside Act 1981 (Natural England 2018, JNCC 2019) and support large populations of old-growth and veteran beech (*Fagus sylvatica L. (Fagales: Fagaceae)*). Sample size was determined by the frequency, volume, accessibility, and suitability of rot hole habitats from which material could be collected (7, 5, and 16 trees at Epping, Savernake, and Windsor, respectively). Visual searches were conducted in pre-selected areas of each woodland determined by availability of fallen trees for extraction of cross-sections for a linked mycological project. Trees within a ~25 m radius of these areas were investigated, as well as those found on walking routes guided by site managers.

Rot samples were collected from standing and fallen beech trees (felled immediately before sampling except one tree for which the hole was basal). The height of rot holes from the base of trees, tree diameter at breast height (1.3 m above ground), and rot hole opening dimensions were measured (length by width; mm). Between 500 and 2,000 cm^3^ of rot was removed by gloved hand and placed into a plastic zip-lock bag; never more than 50% of the available rot was collected (additional sample collection information is available in the Supplementary Materials). The material was transported to the laboratory where the volume was estimated, and all material stored at 4°C prior to sorting.

On breaking the substrate apart, any invertebrates found were placed in tubes containing 70% ethanol (VWR International, Radnor, PA). All remaining material was processed through Tullgren funnels (Burkard Scientific, Uxbridge, United Kingdom) and left for 72 h with vials containing 70% ethanol used for collection. Invertebrates were identified under an Olympus SZX7 stereomicroscope using morphological keys. While identifications were made to species level when appropriate specimens and morphological keys were available, analyses were conducted at the family level to avoid bias by ensuring even representation of diversity across taxa. The feasibility of species identification was limited by a combination of the lack of appropriate species-level keys (e.g., for Acari), the majority of specimens being immature and thus unidentifiable (e.g., in Annelida), and the poor condition of some collected specimens. While appreciating that species-level identification provides the greatest resolution, the taxonomic breadth covered in this study was considered sufficient to allow informative and valuable analysis.

### Rot Characterization

Each sample was characterized by density (g/cm^3^), water content (%), and water potential (MPa). Density (*d*) and water content (*u*) were determined by weighing, oven-drying (at ~60°C for 72 h) and further weighing, prior to calculating using:


$$\begin{array}{l} d=\frac{m_{dry}}{V} \end{array}$$ (1)

$$\begin{array}{l} m_{w}=\;m_{wet}-\;m_{dry} \end{array}$$ (2a)

$$\begin{array}{l} u=\frac{m_{w}}{m_{wet}} \end{array}$$ (2b)

where *v* is the fresh volume (cm^3^), *m*_*w*_ is the mass of water (g), *m*_wet_ is the wet mass (g), and *m*_dry_ is the oven-dry mass (g).

Water potential was measured using a WP4C Dew Point PotentiaMeter (Decagon Devices, Pullman, WA), calibrated using 0.5 M KCl standard solution (Labcell Ltd., Alton, United Kingdom; additional water potential determination information is available in Supplementary Materials. The PotentiaMeter was set to precise mode and the set-point temperature was 25°C.

### Statistical Analysis

All analyses were completed using ‘R’ statistical software version 3.4.4 (R Core Team 2015; additional statistical analysis information is available in Supplementary Materials). To account for the different sample volumes, invertebrate abundances in each sample were converted into relative standardized abundances (percentages of the total sample community). Initial exploratory analyses were completed following the steps outlined in Zuur et al. (2010), including checks for normality, heteroscedasticity, and outliers. Throughout, the accuracy and validity of models and statistical tests were assessed following Thomas *et al.* (2015) and Zuur et al. (2007).

To address the first hypothesis and understand the different structure of invertebrate communities within and across woodland sites, we described the invertebrate community structure in rot holes using several methods. Firstly, abundance, richness, and diversity were calculated. The latter was summarized at three scales (Hill 1973): rot holes (α), woodlands (β), and across all woodland sites (γ). Simpson’s diversity index was used to calculate α-diversity (Hulbert 1971), while β-diversity was calculated from Bray–Curtis distances using the ‘betapart’ package (Baselga et al. 2018), providing information on balanced variation (nesting), abundance gradient components of dissimilarity (turnover), and the sum of these values (Baselga 2017). γ-Diversity was calculated across woodlands, as described by Jost (2006, 2007). Variation in these metrics within and between sites was assessed using Kruskal–Wallis rank sum tests (Ruxton and Beauchamp 2008). Secondly, the structure of invertebrate communities was characterized using nonmetric multidimensional scaling (Kruskal 1964a, b). This was computed using the Jaccard Index (Jaccard 1908), with square root transformation and Wisconsin double standardization (Kenkel and Orloci 1986). We also used multivariate generalized linear models (M-GLMs) to assess the differences in the community composition between sites (Warton et al. 2012). Thirdly, nonrandom patterns of invertebrate co-occurrence were assessed using the ‘co-occur’ package (Griffith et al. 2016). A matrix of taxonomic family presence–absence across sites was compared to randomized matrices (*n* = 1,000) to identify either positive or negative associations between families (Veech 2013). This allowed an understanding of the co-occurrence of families across all sites and woodlands. Finally, nestedness across invertebrate communities, the ordered loss of families across sites or ecological gradients, was assessed using the binary-matrix nestedness temperature calculator (BINMATNEST; Atmar and Patterson 1993, Rodríguez-Gironés and Santamaria 2006). A presence–absence matrix was used, reordering rows and columns to maximize family nestedness and calculate ‘temperature’ (0–100°C), an index of the deviation from perfect nestedness where 100°C is perfectly nested. A Monte-Carlo approach was used to assess statistical significance, with 1,000 randomly generated matrices (null-model III; Heino et al. 2009) compared to the presence–absence matrix.

To address the two specific hypotheses related to habitat characteristics and conditions, invertebrate community structure (using the methods described above) was then related to rot hole environmental characteristics. We first summarized the environmental conditions measured in the rot hole habitats, using principal component analysis (Abdi and Williams 2010). Following this, univariate metrics of invertebrate community structure (e.g., abundance, richness, and diversity) were generated using GLMs, with Gaussian distributions and identity link functions. We also used Poisson M-GLMs, constructed using the ‘mvabund’ package (Wang et al. 2012), to assess variation between rot hole habitats and relationships between community structure and environmental conditions for multivariate community data (i.e., site × taxon matrix).

## Results

### Invertebrate Diversity in Rot Holes

A total of 9,577 individuals from 66 invertebrate families were collected (Supplementary Tables S1 and S2). Communities mostly comprised common taxa. Several saproxylic specialist taxa were present, including click (Elateridae) and feather-winged (Ptiliidae) beetles, but comprised a small proportion of taxa (6.4%, mean 14 individuals per tree across seven families).

To test the first hypothesis, we investigated the diversity of rot hole invertebrate communities across woodlands, which was similar (χ ^2^ = 0.13, df = 2, *P* = 0.94; Kruskal–Wallis rank sum test), and communities generally exhibited high α-diversity (0.68 ± 0.17 SE). The diversity of rot holes, at the site (β-diversity) and landscape (γ-diversity) scales, was also high (3.35 ± 0.11 SE and 10.50 ± 0.39 SE, respectively). Differences between diversity across sites, β-diversity (0.96), were predominantly driven by turnover (0.88) rather than nestedness (0.08). BINMATNEST results also showed limited nestedness across rot holes (16.6°C, *P* < 0.05).

### Community Structure of Rot Hole Invertebrates

**Fig. 1.**
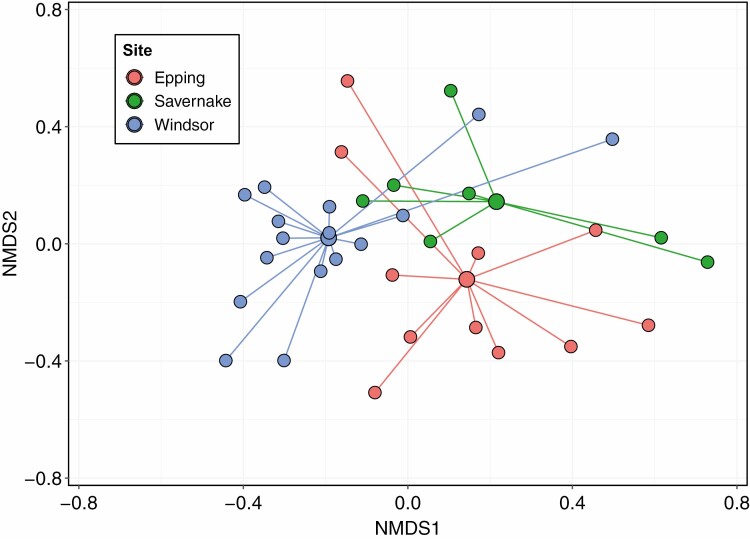
Nonmetric multidimensional scaling of invertebrate community structure in rot holes. Larger central nodes are mean coordinates for the invertebrate community at each woodland site, with radiating nodes indicating the communities of each rot hole across the three woodlands (Epping, Savernake, and Windsor Forests). Separation of nodes on both axes indicates Jaccard dissimilarity between communities.

**Fig. 2.**
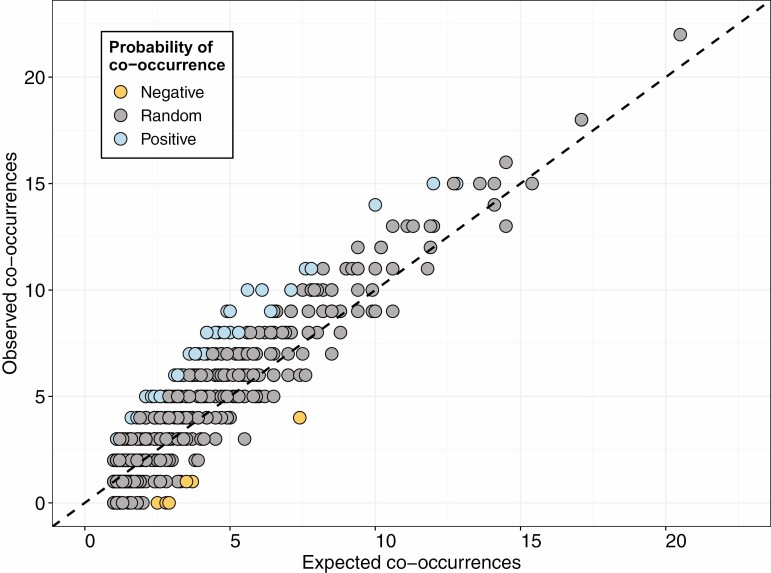
Probability of co-occurrence for invertebrate taxa in rot holes from co-occurrence analysis. Blue and yellow icons indicate nonrandom positive and negative interactions, respectively.

Invertebrate communities varied between rot holes and woodlands. Although variation within sites was high (Fig. 1), community structure was significantly different between the three woodlands (*R*^2^ = 0.09, *W* = 66.09, *P* < 0.001; M-GLM). Co-occurrence was infrequent across rot holes, with 71.91% of taxa combinations occurring once or less (*n* = 1,590). In the subset of co-occurrences (*n* = 621), only 7.7% (*n* = 48) were nonrandom, with most (*n* = 42) of these combinations being positive (Fig. 2; Supplementary Table S3).

### Influence of Rot Hole Habitat Characteristics on Invertebrate Communities

**Fig. 3.**
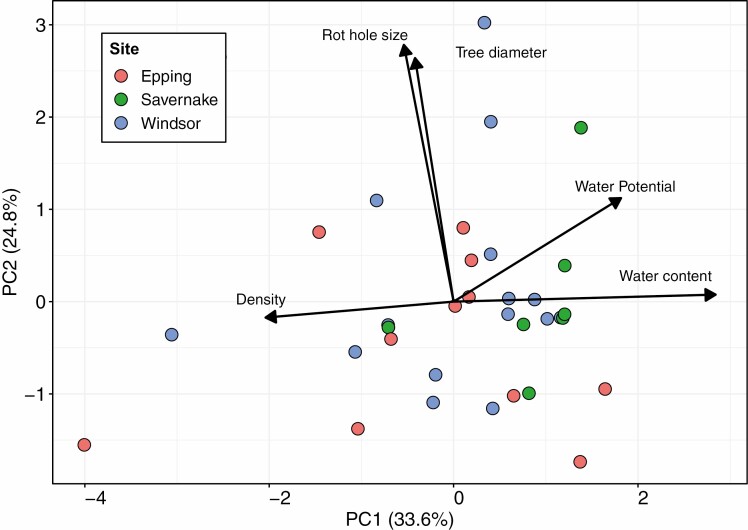
Principal component analysis results for environmental variables measured across rot holes. Arrow length indicates the relative strength of relationship between PCs and environmental variables (i.e., loadings).

Habitat characteristics showed a large degree of variation (Supplementary Table S4). The first two PCs explained 58.3% of variation in rot hole characteristics and environmental conditions (Fig. 3). PC1 explained 33.6% of variation and was negatively related to rot density (−0.51), but positively to water content (0.71) and water potential (0.45). PC2 explained 24.8% of variation and was positively related to tree diameter (0.66) and rot hole dimensions (0.69).

To address the second and third hypotheses, we compared invertebrate communities against the characteristics of the rot holes they inhabited. Invertebrate community composition was significantly related to PCs (*R*^2^ = 0.13, *t*_2,31_ = 81.65, *P* < 0.001; M-GLM). Communities were different from one another along both PC1 (moisture and density; *t*_1,32_ = 56.98, *P* < 0.001) and PC2 (dimensions of the rot hole and diameter of the tree; *t*_1,32_ = 42.58, *P* < 0.001); the most distinct pattern was lower richness and abundance present in the wettest and least dense rot hole habitats. The nestedness of communities was related to neither PC (*R*^2^ = 0.09, *F*_2,31_ = 2.55, *P* = 0.18).

## Discussion

Rot hole invertebrate communities were diverse and heterogeneous, with considerable differences in community structure and biodiversity within habitats (α), between sites (β), and across the landscape (γ). Variation in community structure was driven by a range of environmental conditions, including water content and substrate density, as well as rot hole dimensions and tree diameter. Heterogeneity of rot holes is thus an important consideration in the management and conservation of these woodland microhabitats, even at the family level.

The rot hole communities were very diverse, comprising a variety of families involved directly and indirectly in the decomposition process (Irmler et al. 1996, A’Bear et al. 2013, Gibb et al. 2013, Ulyshen 2016). The difference in communities between different rot densities and water contents, and the effect of these characteristics on diversity, indicates the importance of sustaining a consistent turnover of rot hole habitats to provide habitats at different stages of the decomposition process.

Community diversity was primarily driven by intersite variation and environmental gradients both between sites and across rot holes. Climatological and geographical differences along a gradient from west to east (Savernake, Windsor, and Epping), as well as differences in the regional invertebrate families, are likely to be responsible for generating systematic differences in community structure between woodlands (e.g., drier warmer conditions in the south-east may promote the greater diversity of spiders at Epping Forest; Messas et al. 2014, Souza et al. 2015). Although there were differences between woodland sites, at finer spatial scales, biotic and abiotic processes were responsible for greater variation in invertebrate community composition. Biotic processes such as community assembly influence richness and community composition of fungi and invertebrates (Dickie et al. 2012, Ottosson et al. 2014, Fukami 2015, Hiscox et al. 2015) but in the present study, abiotic conditions appear predominantly to influence rot hole community composition and diversity.

Abiotic conditions directly affect invertebrate community composition due to the specific habitat requirements of many taxa (Quinto et al. 2014, Faccoli et al. 2016, Schauer et al. 2018). Both moisture and substrate density are known to influence the colonization of rot holes by obligate saproxylic taxa such as the scarabid chafer, *Gnorimus nobilis (Linnaeus, 1758; Coleoptera: Scarabaeidae)* (Windsor 2014). These abiotic conditions also affect fungal colonization which subsequently, through the process of inhibitory or facilitative effects, alters the suitability of the substrate for invertebrate taxa (Kaila et al. 1994, Fukami et al. 2010, Weslien et al. 2011, Jacobsen et al. 2015).

Although the conservation of veteran trees has been identified as important for woodland management (Rose et al. 2001, Stahlheber et al. 2015), an understanding of how rot hole heterogeneity influences the structure of inhabiting communities is vital to guide this (Schiegg 2000, Ulyshen 2016, Lindenmayer and Laurance 2017). The findings of this study signpost several important considerations for future management and conservation activities. By highlighting the importance of microhabitat heterogeneity, arguably above habitat quantity, in determining woodland biodiversity, management decisions concerning the removal of large old trees may prioritize the maintenance of those trees providing the most heterogeneous microhabitats. Management plans should focus not just on retaining rot hole habitats but maintaining a high diversity of rot hole characteristics and conditions to optimize biodiversity.

## Supplementary Material

ieab071_suppl_Supplementary_Materials
