# Supplementary material for: Influence of European Beech (Fagales: Fagaceae) Rot Hole Habitat Characteristics on Invertebrate Community Structure and Diversity
Source: J Insect Sci. 2021 Sep 23;21(5):7. doi: 10.1093/jisesa/ieab071 (PMC8458099; doi:10.1093/jisesa/ieab071)
Supplement: ieab071_suppl_Supplementary_Materials [file ieab071_suppl_supplementary_materials.docx]

*Supplementary Materials for*

**Influence of European beech (*Fagus sylvatica*) rot hole habitat characteristics on invertebrate community structure and diversity**

**Study sites**

Samples were collected from three ancient woodland sites in the south of England: Epping Forest (51°39’24.7” N, 0°02’33.0” E), Savernake Forest (51°23’39.4” N, 1°41’07.1” W) and Windsor Forest (51°26’02.5” N, 0°38’37.2” W), in February to July 2016. These sites were selected for the presence of large populations of old-growth and veteran beech (*Fagus sylvatica*) trees situated among other tree species (e.g. oak, ash, sycamore). All three sites are Sites of Special Scientific Interest (SSSI) notified under Section 28 of the Wildlife and Countryside Act 1981 (English Nature 1990). The number of trees sampled was determined by the frequency, volume, accessibility and suitability of rot habitats from which material could be collected. Across the study, rot samples were taken from 7, 5 and 16 trees at Epping, Savernake and Windsor Forests, respectively. Those trees sampled in Windsor Forest were also included in a study concerning the habitat of the violet click beetle *Limoniscus violaceus* (Cuff et al. 2020)

Windsor Forest and Great Park covers an area of 1,777 ha and is renowned for its provision of habitat for several rare species; it is a designated SAC (Special Area of Conservation; Speight 1989; JNCC 2019b). The beech trees in Windsor Forest are often veteran, many possessing large hollows, affording habitat for many saproxylic invertebrates (JNCC 2019b). Site-improvement plans emphasise maintenance of veteran beech trees to sustain populations of rare invertebrates, such as the violet click beetle, *Limoniscus violaceus* (Müller, 1821). Epping Forest covers 1,728 ha and is a recognised SAC due to its populations of rare saproxylic invertebrates, for example, the stag beetle, *Lucanus cervus* (English Nature 1990; JNCC 2019a). Finally, Savernake Forest covers 905 ha and, until recently, was closed to the public, resulting in fewer species records being available from the site. The forest comprises a large area of continuous ancient woodland with a large diversity of saproxylic invertebrate species, many of which are rare (Colquhoun 1941).

**Sample collection and sorting**

Samples were collected between February and July 2016. Rot samples were taken from accessible rot holes on both standing and fallen beech trees. Fallen trees included those that had fallen naturally, as well as those that had been felled. The volume of material sampled from a rot hole varied slightly with availability and accessibility. Approximately 500–2,000 cm^3^ of rot was removed, but at no time was more than 50% of the available rot collected. The samples taken were proportional to the total material available. The height of rot holes from the base of trees, tree diameter at breast height (DBH; 1.3 m above ground), and rot hole opening dimensions were measured (length by width; mm). The material was then transported back to the laboratory at Cardiff University on the same day for sorting and analysis; the volume collected was estimated and all material stored at 4 °C prior to sorting.

Samples from rot holes were placed in foil trays and visually examined for invertebrates. The rot material was broken apart and any invertebrates found were placed in universal tubes (20 x 90 mm; 25 ml; Sarstedt, Nümbrecht, Germany) containing 70% ethanol (VWR International, Radnor, PA, USA). All remaining material was placed in Tullgren funnels (Burkard Scientific, Uxbridge, UK) and left for 72 hours; invertebrates present were collected in universal tubes containing 70% ethanol. Invertebrates were identified under an Olympus SZX7 stereomicroscope using the following morphological keys: AIDGAP: Key to the Earthworms of the UK and Ireland (Sherlock 2012); Collins’ Field Guide: Spiders of Britain and Northern Europe (Roberts 1996); AIDGAP: A Key to the Woodlice of Britain and Ireland (Hopkin 2012); AIDGAP: Key to the identification of British Centipedes (Barber 2008); Naturalists’ Handbooks 24: Ants (Skinner and Allen 1996); AIDGAP: A Key to the Major Groups of British Terrestrial Invertebrates (Tilling 1987); An Introduction to Centipedes, Millipedes and Woodlice (Richards 2011); Beetles of Britain and Ireland Volume 1 (Duff 2012); An Introduction to the Immature Stages of British Flies (Smith 1989) and Illustrated Key to the British False Scorpions (Pseudoscorpions) (Legg and Farr-Cox 2016). While identifications were made to species level when appropriate specimens and morphological keys were available, analyses were conducted at the family level to avoid bias and ensure even representation of diversity across taxa since this resolution was achieved for all specimens. This was due to a lack of species-level data for several groups due to a lack of species-level keys (e.g. for Acari), the majority of specimens being immature and thus unidentifiable (e.g. in Annelida), or poor condition of some collected specimens. While species-level identification provides the greatest resolution, the taxonomic breadth covered in this study renders even family-level analysis informative and valuable.

**Rot characterisation**

Water potential of rot samples was determined in a Decagon Devices WP4C Dew Point PotentiaMeter (Decagon Devices, Pullman, WA, USA), calibrated using 0.5 M KCl standard solution (Labcell Ltd., Alton, UK). Water potential, the measure of availability of water, was investigated given its importance to many organisms, notably the fungi upon which many of the focal invertebrates likely depend. The rot sample was placed in a stainless-steel sample cup (15 ml) with the base of the cup entirely covered and no more than half-filled. The sample was incubated on an AquaTherm temperature equilibrium plate (Decagon Devices) at approximately 24 °C; in this way, the temperature difference between the sample and the chamber block was minimalised. The PotentiaMeter was set to precise mode and the set-point temperature was set at 25 °C. Water potential was calculated using:

1. $\varphi= \frac{RT}{M} \cdot ln\left( \frac{\rho}{\rho_{o}} \right)$

where *Ψ* is the rot water potential (MPa), *R* is the gas constant (8.31 J mol K^-1^), *T* is the temperature (K) of the sample, *M* is the molecular mass of water (18.02 g mol^-1^), *ρ* is the vapour pressure of the air, and *ρ_o_* is the saturation vapour pressure at sample temperature.

**Statistical analysis**

All analyses were completed using ‘R’ statistical software version 3.4.4 (R Core Team, 2015). Initial exploratory analysis was completed for all data to prevent common statistical problems, with the methods following those proposed by Zuur *et al.* (2010). Throughout the analysis, the accuracy and validity of models and statistical tests were assessed following Thomas *et al.* (2015) and Zuur *et al.* (2007). Briefly, residual normality was assessed using quantile-quantile (QQ) plots, homogeneity of variance was determined by plotting residuals against fitted values, and influential observations were investigated using Cook’s leverage distances.

Invertebrate communities were characterised using a range of biological metrics, including abundance, species richness and diversity. The latter was summarised at three scales (Hill, 1973): rot holes (α), woodland sites (β) and across all woodland sites (γ). Firstly, the Simpson’s diversity index (SI) was used to calculate α-diversity (Hulbert 1971). Secondly, β-diversity was calculated for abundance data using Bray-Curtis indices using the ‘betapart’ package (Baselga *et al.* 2018), allowing an understanding of the levels of nestedness and turnover present within rot hole communities (Baselga, 2017). This method accounts for balanced variation (nesting), abundance gradient components of dissimilarity (turnover) and the sum of both of these values (total abundance-based dissimilarity; Baselga, 2017). Finally, γ-diversity was calculated for the rot hole communities present across the three woodlands, as described by Jost (2006; 2007). Variation in biological metrics and diversity measures between sites were assessed using Kruskal-Wallis rank sum tests (Ruxton & Beauchomp, 2008).

The taxonomic structure of invertebrate communities in rot holes was characterised using non-metric multidimensional scaling (NMDS; Kruskal, 1964a; 1964b), coupled with multivariate generalised linear models (M-GLMs; Warton *et al.* 2012). NMDS was computed using the Jaccard Index (Jaccard, 1908), with a square root transformation and Wisconsin double standardisation to account for the influence of both common and rare taxa across communities (Kenkel & Orloci, 1986). Poisson M-GLMs, constructed using the “mvabund” package (Wang *et al.* 2012), were used to assess taxonomic variations between rot holes as well as relationships between environmental conditions and community structure.

Non-random patterns of invertebrate taxa co-occurrence were assessed using presence-absence matrices and completed using the “co-occur” package (Griffith *et al*. 2016). The method used here calculates the probability that two species would co-occur more or less frequently than if the two species were distributed independently across sites (Veech, 2013). The matrix of species presence-absence is compared to randomised matrices (n = 1,000) to determine whether communities are structured, with either positive or negative associations between species (Veech, 2013). Specific combinations of taxa were subsequently interrogated to identify likely determinants of community assembly within rot holes across woodlands.

The level of nestedness across rot hole invertebrate communities was assessed using the binary-matrix nestedness temperature calculator (BINMATNEST; Atmar & Patterson, 1993; Rodríguéz & Santamaria, 2006). A species presence-absence matrix was used, reordering rows and columns to maximise species nestedness and calculate temperature (0–100 °C). The temperature is an index of the deviation of the matrix from perfect nestedness. As an example, the two extremes of the temperature correspond to: (i) perfectly nested matrices, where rare species occur only in the most species rich communities (*T* = 100 °C); and (ii) random matrices where rare species are distributed across matrices (*T* = 0 °C). A Monte-Carlo approach was used to assess the statistical significance of the temperature value, with 1,000 randomly generated matrices compared to the presence-absence matrix. The conservative null-model III was used in this study given its lack of sensitivity to species richness and variable occurrence (Heino *et al.* 2009). The order or rank of the sites in the matrix (based on the level of nestedness) can be compared to independent correlates, in this case environmental conditions, to understand the likely drivers of observed species nestedness. Here, the relationships between nestedness ranks and environmental variables were assessed using generalised linear models (GLMs), with gaussian distributions and identity link functions.

Finally, to understand the influence of environmental conditions on invertebrate communities occupying rot holes, the suite of environmental characteristics measured within each rot hole (see *Rot characterisation* above) was first summarised using Principal Components Analysis (PCA; Abdi & Williams, 2010). This eigenvalue-based method uses orthogonal transformations to convert variables into a set of independent variables called principal components (PCs). The PCs derived from the ordination of environmental variables were subsequently related to community structure, species nestedness and biological metrics using a combination of M-GLMs and non-parametric linear regression.

**References:**

Abdi, H. and Williams, L.J. (2010). Wiley Interdisciplinary Reviews: Principal component analysis. *Computational Statistics* **2**: 433–459.

Atmar, W. and Patterson, B.D. (1993). The measurement of order and disorder in the distribution of species in fragmented habitat. *Oecologia* **96**: 373–382.

Baselga, A. (2017). Partitioning abundance-based multiple-site dissimilarity into components: balanced variation in abundance and abundance gradients*. Methods in Ecology and Evolution* **8**: 799-808.

Baselga, A., Orme, D., Villeger, S., De Bortoli, J., and Leprieur, F. (2018). betapart: Partitioning beta diversity into turnover and nestedness components. R package version 1.5.1. <https://CRAN.R-project.org/package=betapart>.

Barber, A.D. 2008. *Key to the Identification of British Centipedes*. Shrewsbury, UK: FSC Publications.

Colquhoun, M.K. (1941). The birds of Savernake Forest, Wiltshire. *Journal of Animal Ecology* **10**:2 5–34.

Cuff, J.P., Müller, C.T., Gilmartin, E.C., Boddy, L.E. and Jones, T.H. 2020. Home is where the heart rot is: violet click beetle, Limoniscus violaceus (Müller , 1821), habitat attributes and volatiles. *Insect Conservation and Diversity* , p. in press. doi: 10.1111/icad.12441.

Duff, A.G. 2012. *Beetles of Britain and Ireland Volume 1: Sphaeriusidae to Silphidae*. West Runton, UK: A.G. Duff Publishing.

English Nature (1990). *Designated Sites: Epping Forest/London Boroughs of Waltham Forest and Redbridge*. English Nature pp. 1–4

Griffith, D.M., Veech, J.A. and Marsh, C.J. (2016). cooccur: Probabilistic species co-occurrence analysis in R. *Journal of Statistical Software* **69**: 1–17.

Heino, J., Mykra, H. and Muotka, T. (2009). Temporal variability of nestedness and idiosyncratic species in stream insect assemblages. *Diversity and Distributions* **15**: 198–206.

Hill, M. (1973). Diversity and evenness: A unifying notation and its consequences. *Ecology* **54**: 427–432.

Hopkin, S. 2012. *A key to the woodlice of Britain and Ireland*. 1st ed. Telford, UK: FSC Publications.

Hulbert, S.H. (1971). The non-concept of species diversity: a critique and alternative parameters. *Ecology* **52**: 577–586.

Jaccard, P. (1908). Nouvelles recherches sur la distribution florale. *Bulletin Société Vaudoise des Sciences Naturelles* **44**: 223–270.

JNCC (2019a). *Epping Forest* [Online]. Available at: <http://jncc.defra.gov.uk/protectedsites/sacselection/sac.asp?EUcode=UK0012720> [Accessed: 4 February 2019].

JNCC 2019b. Windsor Forest and Great Park [Online]. Available at: http://jncc.defra.gov.uk/ProtectedSites/SACselection/sac.asp?EUCode=UK0012586 [Accessed: 4 February 2019].

Jost, L. (2006). Entropy and diversity. *Oikos* **113:** 363–375.

Jost, L. (2007). Partitioning diversity into independent alpha and beta components. *Ecology* **88:** 2427–2439.

Kenkel, N.C. and Orloci, L. (1986). Applying metric and nonmetric multidimensional scaling to ecological studies: Some new results. *Ecology* **67**: 919–928.

Kruskal, J.B. (1964a). Multidimensional scaling by optimizing goodness-of-fit to a nonmetric hypothesis. *Psychometrika* **29**: 1–28.

Kruskal, J.B. (1964b). Nonmetric multidimensional scaling: a numerical method. *Psychometrika* **29**: 115–129.

Legg, G. and Farr-Cox, F. 2016. *Illustrated key to the British false scorpions (pseudoscorpions)*. 1st ed. Telford, UK: FSC Publications.

R Core Team. (2015). *R: A language and environment for statistical computing*. R Foundation for Statistical Computing. Vienna, Austria.

Richards, P. 2011. *An introduction to centipedes, millipedes & woodlice*. 1st ed. Pisces Publications.

Roberts, M.J. 1996. *Spiders of Britain and Northern Europe*. 1st ed. London, UK: HarperCollins Publishers Ltd.

Rodríguez-Gironés, M.A. and Santamaria, L. (2006). A new algorithm to calculate the nestedness temperature of presence-absence matrices. *Journal of Biogeography* **33**: 924–935.

Ruxton, G.D. and Beauchamp, G. (2008). Some suggestions about appropriate use of the Kruskal-Wallis test. *Animal Behaviour* **76**: 1083–1087.

Sherlock, E. 2012. *Key to the earthworms of the UK and Ireland*. 1st ed. Telford, UK: FSC Publications.

Skinner, G.J. and Allen, G.W. 1996. *Naturalists’ Handbook 24: Ants*. 1st ed. Slough, UK: The Richmond Publishing Co. Ltd.

Smith, K.G.V. 1989. *An introduction to the immature stages of British flies: Diptera larvae, with notes on eggs, puparia and pupae*. 1st ed. London, UK: Royal Entomological Society of London.

Speight, M.C.D. (1989). Life in dead trees - a neglected part of Europe wildlife heritage. *Environmental Conservation* **16**: 354–356.

Thomas, R., Lello, J., Medeiros, R., Pollard, A., Seward, A., Smoth, J., Vafidis, J. and Vaughan, I. (2015). *Data analysis with R statistical software: A guidebook for Scientists*. Eco-Explore, Newport, UK.

Tilling, S.M. 1987. *A key to the major groups of British terrestrial invertebrates*. 1st ed. Telford, UK: FSC Publications.

Veech, J.A. (2013). A probabilistic model for analysing species co-occurrence. *Global Ecology and Biogeography* **22**: 252–260.

Wang Y., Neuman U., Wright S. and Warton D. I. (2012). mvabund: an R package for model-based analysis of multivariate abundance data. *Methods in Ecology and Evolution* **3**: 471–473.

Warton D. I., Wright S. and Wang, Y. (2012). Distance-based multivariate analyses confound location and dispersion effects*. Methods in Ecology and Evolution* **3**: 89–101.

Zuur, A.F., Leno, E.N. and Elphick, C.S. (2010). A protocol for data exploration to avoid common statistical problems. *Methods in Ecology and Evolution* **1**: 3–14.

Zuur, A.F., Leno, E.N. and Smith, G.M. (2007). *Analysing Ecological Data.* Springer, New York, USA.

**Table S1. Relative abundance (mean number of individuals per rot hole) of invertebrate taxa identified at Epping Forest, Savernake Forest and Windsor Forest.**

| **Taxonomic group** | **Abundance (mean (standard error))** | | | **Count (n)** |
| --- | --- | --- | --- | --- |
|  | **Epping** | **Savernake** | **Windsor** | **Total** |
| **Haplotaxida** |  |  |  |  |
| Lumbricidae | 14.3 (6.1) | 16.7 (7.1) | 1.5 (0.6) | 298 |
| **Isopoda** |  |  |  |  |
| Porcellionidae | 51.6 (45.0) | 30.4 (20.1) | 2.8 (1.0) | 825 |
| Trichoniscidae | 0.2 (0.2) | - | 22.2 (14.0) | 357 |
| Oniscidae | 2.7 (2.5) | 0.6 (0.4) | 5.8 (2.0) | 127 |
| **Araneae** |  |  |  |  |
| Amaurobiidae | 1.7 (1.4) | - | 0.1 (0.1) | 20 |
| Agelenidae | 0.2 (0.2) | - | - | 2 |
| Dysderidae | 6.1 (5.5) | - | - | 67 |
| Clubionidae | 1.4 (1.3) | - | - | 15 |
| Philodromidae | 0.4 (0.3) | - | - | 4 |
| Oonopidae | - | - | 0.1 (0.1) | 1 |
| Linyphiidae | 0.1 (0.1) | - | 0.1 (0.1) | 2 |
| Thomisidae | 0.2 (0.2) | - | - | 2 |
| **Hymenoptera** |  |  |  |  |
| Formicidae | - | - | 0.1 (0.1) | 2 |
| **Coleoptera** |  |  |  |  |
| Curculionidae | 15.9 (12.0) | 1.7 (1.4) | 0.8 (0.4) | 199 |
| Coccinellidae | 0.1 (0.1) | - | - | 1 |
| Staphylinidae | 0.8 (0.4) | 3.9 (1.9) | 1.8 (0.5) | 64 |
| Elateridae | 8.0 (6.9) | 0.7 (0.5) | 0.6 (0.3) | 103 |
| Tenebrionidae | 3.2 (3.0) | - | 1.6 (1.2) | 61 |
| Apionidae | 0.1 (0.1) | - | - | 1 |
| Dermestidae | - | - | 0.1 (0.1) | 1 |
| Carabidae | 0.6 (0.4) | 1.7 (1.2) | 0.5 (0.2) | 26 |
| Ptiliidae | 1.2 (0.5) | 3.3 (1.6) | 6.4 (3.2) | 139 |
| Byrrhidae | 0.3 (0.1) | 1.4 (0.9) | 0.8 (0.3) | 25 |
| **Scolopendromorpha** |  |  |  |  |
| Cryptopidae | 0.4 (0.1) | - | 1.2 (0.4) | 23 |
| **Julida** |  |  |  |  |
| Julidae | 0.2 (0.1) | 12.4 (7.0) | 0.5 (0.3) | 97 |
| **Geophilomorpha** |  |  |  |  |
| Geophilidae | - | 4.9 (4.3) | 0.3 (0.2) | 39 |
| Himantariidae | - | - | 0.3 (0.1) | 4 |
| Schendylidae | - | - | 0.1 (0.1) | 2 |
| **Lithobiomorpha** |  |  |  |  |
| Lithobiidae | - | - | 0.6 (0.3) | 9 |
| **Polydesmida** |  |  |  |  |
| Polydesmidae | 0.1 (0.1) | - | - | 1 |
| **Diptera** |  |  |  |  |
| Calliphoridae | 0.8 (0.5) | 0.3 (0.2) | 0.5 (0.2) | 19 |
| Stratiomyidae | 5.3 (2.9) | 0.3 (0.3) | 1.5 (0.7) | 84 |
| Muscidae | 0.1 (0.1) | - | - | 1 |
| Mycetophilidae | 20.5 (14.1) | 30.1 (25.9) | 2.2 (0.9) | 471 |
| Trichoceridae | 11.2 (6.4) | 52.1 (35.2) | 0.4 (0.2) | 495 |
| Tipulidae | 0.6 (0.5) | 8.6 (5.2) | 2.4 (1.7) | 105 |
| Syrphidae | 5.6 (4.5) | - | - | 4 |
| Ceratopogonidae | - | - | 0.2 (0.2) | 3 |
| Dolichopodiidae | - | 1.7 (1.6) | 0.4 (0.3) | 18 |
| Bibionidae | 3.5 (3.3) | 5.6 (5.2) | - | 77 |
| Sciaridae | 0.2 (0.1) | - | - | 2 |
| Chironomidae | 2.2 (1.8) | 0.3 (0.3) | 0.1 (0.1) | 27 |
| **Orbatida** |  |  |  |  |
| Euphthiracaridae | - | - | 2.6 (2.4) | 42 |
| Phthiracaridae | 0.4 (0.2) | 0.4 (0.4) | 1.3 (0.4) | 27 |
| Malaconothridae | - | - | 0.2 (0.2) | 3 |
| Ceratozetidae | - | - | 0.1 (0.1) | 1 |
| Nothridae | - | - | 0.3 (0.2) | 4 |
| Crotoniidae | 1.5 (1.4) | - | 0.1 (0.1) | 18 |
| **Mesotigmata** |  |  |  |  |
| Veigaiidae | 42.9 (21.6) | 71.6 (50.1) | 56.9 (11.2) | 1884 |
| Epicriidae | - | - | 0.1 (0.1) | 1 |
| Uropodidae | 1.5 (1.1) | 20.1 (16.8) | 29.4 (17.6) | 628 |
| **Trombiformes** |  |  |  |  |
| Anystidae | 0.2 (0.2) | - | - | 2 |
| **Sarcoptiformes** |  |  |  |  |
| Achipteriidae | 0.6 (0.4) | 24.0 (22.2) | 2.1 (0.7) | 207 |
| **Parasitiformes** |  |  |  |  |
| Protodynichidae | 9.2 (8.8) | - | - | 101 |
| **Hemiptera** |  |  |  |  |
| Reduviidae | 7.3 (4.4) | 0.6 (0.5) | 0.1 (0.1) | 85 |
| **Collembola** |  |  |  |  |
| Tomoceridae | 2.7 (1.7) | 13.4 (10.3) | 2.1 (0.7) | 158 |
| Onychiuridae | 8.3 (4.4) | 210.7 (181.9) | 34.9 (12.2) | 2124 |
| Spinothecidae | - | - | 0.1 (0.1) | 1 |
| Actaletidae | 9.0 (4.8) | 3.3 (3.0) | 11.3 (6.1) | 303 |
| Paronellidae | 0.5 (0.2) | - | - | 5 |
| **Pseudoscorpionida** |  |  |  |  |
| Chernitidae | 2.5 (1.4) | 1.9 (1.3) | 1.2 (0.4) | 59 |
| Cthoniidae | - | - | 1.1 (0.2) | 17 |
| **Gastropoda** |  |  |  |  |
| Oxychilidae | 0.1 (0.1) | 0.3 (0.2) | 0.8 (0.3) | 16 |
| Discidae | - | - | 0.1 (0.1) | 1 |
| Limacidae | 0.6 (0.6) | - | 0.1 (0.1) | 8 |
| Helicidae | - | - | 0.1 (0.1) | 1 |

**Table S2. Relative abundance (mean number of individuals per rot hole) of invertebrate species identified at Epping Forest, Savernake Forest and Windsor Forest. These groups were identified to species level given the high availability of identifiable (i.e. intact adults where necessary) specimens for which appropriate taxonomic keys were readily available. Analyses were conducted at family level to ensure even representation of diversity across taxa.**

| **Taxonomic group** | **Abundance (mean (standard error))** | | |
| --- | --- | --- | --- |
|  | **Epping** | **Savernake** | **Windsor** |
| **Lumbricidae** |  |  |  |
| *Dendrodrilus rubidus* Savigny, 1826 | 1.6 (2) | 1.8 (0.7) | 0 (0) |
| *Eisenia fetida* (Savigny, 1826) | 9.2 (9) | 1.1 (10.3) | 0.4 (0.9) |
| *Lumbricus castaneus* (Savigny, 1826) | 0.3 (0.2) | 0 (0) | 0 (0) |
| **Trichoniscidae** |  |  |  |
| *Haplophthalmus danicus* Budde-Lund, 1879 | 0.3 (0.2) | 0 (0) | 8.5 (22) |
| *Trichoniscus pusillus* Brandt, 1833 | 0 (0) | 0 (0) | 0.1 (0.1) |
| **Porcellionidae** |  |  |  |
| *Porcellio scaber* Latreille, 1804 | 8.5 (51.6) | 7.5 (30.4) | 2.3 (2.8) |
| **Oniscidae** |  |  |  |
| *Oniscus asellus* Linnaeus, 1758 | 0.2 (2.7) | 0 (0.6) | 3.3 (5.8) |
| **Chernitidae** |  |  |  |
| *Chernes cimicoides* (Fabricius, 1793) | 0.5 (2.5) | 0.3 (1.9) | 1.1 (1.1) |
| **Cthoniidae** |  |  |  |
| *Chthonius ischnocheles* (Hermann, 1804) | 0 (0) | 0 (0) | 0.6 (1.1) |
| **Cryptopidae** |  |  |  |
| *Cryptops hortensis* (Donovan, 1810) | 0.3 (0.3) | 0 (0) | 0.5 (1.2) |
| **Geophilidae** |  |  |  |
| *Geophilus truncorum* Bergsoë & Meinert, 1886 | 0 (0) | 0.8 (5.1) | 0.2 (0.3) |
| **Himantariidae** |  |  |  |
| *Stigmatogaster subterranea* (Shaw, 1789) | 0 (0) | 0 (0) | 0.2 (0.3) |
| **Schendylidae** |  |  |  |
| *Schendyla nemorensis* (Koch, 1837) | 0 (0) | 0 (0) | 0.1 (0.1) |
| **Lithobiidae** |  |  |  |
| *Lithobius variegatus* Leach, 1814 | 0 (0) | 0 (0) | 0.3 (0.6) |

**Table S3. Family co-occurrence across rot holes determined by co-occurrence analysis.** The relationship column indicates the direction of co-occurrence, with ‘+’ and ‘-‘ denoting positive and negative co-occurrences, respectively.

| **Family 1** | **Family 2** | **Observed** | **Expected** | **Probability** | **Relationship** |
| --- | --- | --- | --- | --- | --- |
| Cryptopsidae | Amaurobiidae | 3 | 1.1 | 0.031 | + |
| Amaurobiidae | Trichoceridae | 3 | 1.1 | 0.031 | + |
| Amaurobiidae | Oniscidae | 3 | 1.1 | 0.034 | + |
| Oniscidae | Lithobiidae | 3 | 1.1 | 0.034 | + |
| Tenebrionidae | Chthoniidae | 4 | 1.6 | 0.048 | + |
| Geophilidae | Byrrhidae | 4 | 1.8 | 0.052 | + |
| Trichoniscidae | Oxychilidae | 5 | 2.1 | 0.062 | + |
| Calliphoridae | Tilupidae | 5 | 2.1 | 0.062 | + |
| Oniscidae | Geophilidae | 5 | 2.3 | 0.067 | + |
| Tilupidae | Byrrhidae | 5 | 2.4 | 0.069 | + |
| Lumbricidae | Tenebrionidae | 0 | 2.5 | 0.074 | - |
| Geophilidae | Uropodidae | 5 | 2.6 | 0.078 | + |
| Trichoniscidae | Trichoceridae | 0 | 2.8 | 0.083 | - |
| Calliphoridae | Achipteriidae | 0 | 2.9 | 0.086 | - |
| Oniscidae | Tilupidae | 6 | 3.1 | 0.09 | + |
| Calliphoridae | Trichoceridae | 6 | 3.2 | 0.093 | + |
| Byrrhidae | Achipteriidae | 6 | 3.2 | 0.095 | + |
| Stratiomyidae | Elateridae | 6 | 3.3 | 0.097 | + |
| Mycetophilidae | Geophilidae | 1 | 3.5 | 0.104 | - |
| Tomoceridae | Phthiracaridae | 7 | 3.6 | 0.105 | + |
| Curculionidae | Oxychilidae | 1 | 3.7 | 0.109 | - |
| Elateridae | Chernetidae | 7 | 3.8 | 0.111 | + |
| Chernetidae | Tilupidae | 7 | 3.8 | 0.111 | + |
| Cryptopsidae | Chthoniidae | 7 | 3.9 | 0.114 | + |
| Stratiomyidae | Actaletidae | 7 | 4.1 | 0.121 | + |
| Oniscidae | Tomoceridae | 8 | 4.2 | 0.124 | + |
| Oniscidae | Chthoniidae | 8 | 4.2 | 0.124 | + |
| Calliphoridae | Chernetidae | 7 | 4.2 | 0.125 | + |
| Calliphoridae | Staphylinidae | 8 | 4.5 | 0.132 | + |
| Cryptopsidae | Oniscidae | 8 | 4.6 | 0.135 | + |
| Carabidae | Ptiliidae | 8 | 4.8 | 0.14 | + |
| Cryptopsidae | Stratiomyidae | 9 | 4.9 | 0.145 | + |
| Staphylinidae | Byrrhidae | 9 | 5 | 0.147 | + |
| Staphylinidae | Actaletidae | 8 | 5 | 0.147 | + |
| Porcellionidae | Julidae | 8 | 5.3 | 0.156 | + |
| Ptiliidae | Byrrhidae | 8 | 5.3 | 0.156 | + |
| Trichoniscidae | Onychiuridae | 8 | 5.6 | 0.166 | + |
| Chernetidae | Trichoceridae | 10 | 5.6 | 0.166 | + |
| Oniscidae | Chernetidae | 10 | 6.1 | 0.18 | + |
| Carabidae | Onychiuridae | 9 | 6.4 | 0.187 | + |
| Mycetophilidae | Trichoceridae | 10 | 7.1 | 0.208 | + |
| Onychiuridae | Byrrhidae | 10 | 7.1 | 0.208 | + |
| Curculionidae | Ptiliidae | 4 | 7.4 | 0.218 | - |
| Porcellionidae | Oniscidae | 11 | 7.6 | 0.225 | + |
| Phthiracaridae | Onychiuridae | 11 | 7.8 | 0.228 | + |
| Porcellionidae | Staphylinidae | 14 | 10 | 0.294 | + |
| Staphylinidae | Onychiuridae | 15 | 12 | 0.353 | + |
| Veigaiidae | Uropodidae | 15 | 12.8 | 0.376 | + |

**Table S4. Tree, hollow and rot characteristics for each sampled microhabitat.**

| **Site** | **Date** | **Tree Status** | **Tree Diameter (cm)** | **Hole Height (cm)** | **Hole area (cm^2^)** | **Density (g cm^-3^)** | **Water content (%)** | **Water potential (MPa)** |
| --- | --- | --- | --- | --- | --- | --- | --- | --- |
| Windsor | 31/05/2016 | Standing | 138 | 0 | 6119 | 0.162 | 77.68595 | -0.03 |
| Windsor | 31/05/2016 | Standing | 103 | 0 | 7498 | 0.300 | 57.26496 | -0.12 |
| Windsor | 31/05/2016 | Standing | 113 | 0 | 2345 | 0.230 | 79.12886 | -0.54 |
| Windsor | 31/05/2016 | Standing | 102 | 20 | 399 | 0.194 | 77.70115 | -0.56 |
| Windsor | 31/05/2016 | Fallen | 74 | 0 | 5720 | 0.144 | 80.0554 | -0.01 |
| Windsor | 31/05/2016 | Standing | 85 | 0 | 2700 | 0.162 | 83.99209 | -0.09 |
| Windsor | 31/05/2016 | Standing | 124 | 0 | 14910 | 0.158 | 83.19149 | -0.8 |
| Windsor | 31/05/2016 | Standing | 97 | 0 | 780 | 0.122 | 84.35897 | -0.35 |
| Windsor | 31/05/2016 | Standing | 96 | 0 | 1887 | 0.170 | 54.30108 | -8.02 |
| Windsor | 31/05/2016 | Standing | 103 | 0 | 275 | 0.722 | 36.99825 | -0.88 |
| Windsor | 31/05/2016 | Standing | 85 | 0 | 360 | 0.216 | 63.75839 | -2.64 |
| Windsor | 31/05/2016 | Standing | 68 | 0 | 200 | 0.190 | 68.75 | -0.35 |
| Windsor | 31/05/2016 | Standing | 99 | 0 | 17304 | 0.292 | 22.34043 | -0.01 |
| Windsor | 31/05/2016 | Standing | 104 | 0 | 792 | 0.128 | 71.30045 | -0.09 |
| Windsor | 31/05/2016 | Standing | 100 | 0 | 275 | 0.242 | 49.58333 | -0.43 |
| Windsor | 31/05/2016 | Standing | 79 | 0 | 432 | 0.198 | 66.66667 | -4.95 |
| Savernake | 14/03/2016 | Fallen | 99 | 230 | 54 | 0.188 | 79.34066 | -0.05 |
| Savernake | 14/03/2016 | Fallen | 99 | 347 | 306 | 0.132 | 84.82759 | -0.01 |
| Savernake | 14/03/2016 | Fallen | 99 | 347 | 306 | 0.091 | 37.41846 | -1 |
| Savernake | 14/03/2016 | Fallen | 100 | 454 | 357 | 0.124 | 84.72906 | -0.01 |
| Savernake | 09/05/2016 | Standing | 73 | 11 | 280 | 0.148 | 75.81699 | -0.6 |
| Savernake | 09/05/2016 | Standing | 86 | 0 | 5751 | 0.116 | 87.05357 | -0.07 |
| Savernake | 09/05/2016 | Standing | 149 | 89 | 3626 | 0.066 | 92.08633 | -0.02 |
| Epping | 15/02/2016 | Fallen | 136 | 300 | 0 | 0.248 | 72.80702 | 0 |
| Epping | 15/02/2016 | Fallen | 136 | 34 | 330 | 0.536 | 60.87591 | -0.01 |
| Epping | 15/02/2016 | Fallen | 73 | 13 | 119 | 0.074 | 87.70764 | -0.01 |
| Epping | 15/02/2016 | Fallen | 73 | 300 | 88 | 0.25 | 79.47455 | -0.07 |
| Epping | 15/02/2016 | Standing | 47 | 0 | 213 | 0.148 | 85.76923 | -0.35 |
| Epping | 15/02/2016 | Standing | 101 | 101 | 1633 | 0.274 | 74.24812 | -0.08 |
| Epping | 15/02/2016 | Standing | 101 | 101 | 1633 | 0.241 | 71.77849 | -1.74 |
| Epping | 15/02/2016 | Standing | 101 | 101 | 1633 | 0.236 | 30.84746 | -24.75 |
| Epping | 18/07/2016 | Standing | 117 | 133 | 1188 | 0.27 | 75.80645 | -0.01 |
| Epping | 18/07/2016 | Standing | 97 | 147 | 210 | 0.43 | 67.47353 | -0.65 |
| Epping | 18/07/2016 | Standing | 58 | 196 | 1200 | 0.494 | 60.03236 | -0.31 |
